# Complex Relationships between Occupation, Environment, DNA Adducts, Genetic Polymorphisms and Bladder Cancer in a Case-Control Study Using a Structural Equation Modeling

**DOI:** 10.1371/journal.pone.0094566

**Published:** 2014-04-10

**Authors:** Stefano Porru, Sofia Pavanello, Angela Carta, Cecilia Arici, Claudio Simeone, Alberto Izzotti, Giuseppe Mastrangelo

**Affiliations:** 1 Department of Medical-Surgical Specialties, Radiological Sciences and Public Health, Section of Public Health and Human Sciences, University of Brescia, Brescia, Italy; 2 Department of Cardiac, Thoracic, and Vascular Sciences, Unit of Occupational Medicine, University of Padova, Padova, Italy; 3 Department of Medical-Surgical Specialties, Radiological Sciences and Public Health, Section of Surgical Specialties, University of Brescia, Brescia, Italy; 4 Department of Health Sciences, University of Genoa, Italy/Mutagenesis Unit, IRCCS Hospital-University San Martino Company – IST National Institute for Cancer Research, Genoa, Italy; Oklahoma Medical Research Foundation, United States of America

## Abstract

DNA adducts are considered an integrate measure of carcinogen exposure and the initial step of carcinogenesis. Their levels in more accessible peripheral blood lymphocytes (PBLs) mirror that in the bladder tissue. In this study we explore whether the formation of PBL DNA adducts may be associated with bladder cancer (BC) risk, and how this relationship is modulated by genetic polymorphisms, environmental and occupational risk factors for BC. These complex interrelationships, including direct and indirect effects of each variable, were appraised using the structural equation modeling (SEM) analysis. Within the framework of a hospital-based case/control study, study population included 199 BC cases and 213 non-cancer controls, all Caucasian males. Data were collected on lifetime smoking, coffee drinking, dietary habits and lifetime occupation, with particular reference to exposure to aromatic amines (AAs) and polycyclic aromatic hydrocarbons (PAHs). No indirect paths were found, disproving hypothesis on association between PBL DNA adducts and BC risk. DNA adducts were instead positively associated with occupational cumulative exposure to AAs (p = 0.028), whereas XRCC1 Arg 399 (p<0.006) was related with a decreased adduct levels, but with no impact on BC risk. Previous findings on increased BC risk by packyears (p<0.001), coffee (p<0.001), cumulative AAs exposure (p = 0.041) and *MnSOD* (p = 0.009) and a decreased risk by *MPO* (p<0.008) were also confirmed by SEM analysis. Our results for the first time make evident an association between occupational cumulative exposure to AAs with DNA adducts and BC risk, strengthening the central role of AAs in bladder carcinogenesis. However the lack of an association between PBL DNA adducts and BC risk advises that these snapshot measurements are not representative of relevant exposures. This would envisage new scenarios for biomarker discovery and new challenges such as repeated measurements at different critical life stages.

## Introduction

Tobacco smoking and occupational exposures to aromatic amines (AAs) and polycyclic aromatic hydrocarbons (PAHs) are the major risk factors for bladder cancer (BC) [1], [2]. Moreover increasing evidence suggests a significant influence of genetic predisposition on BC incidence [3], [4].

The formation of reactive metabolites of AAs and PAHs and their binding to DNA to give unrepaired/stable adducts, all modulated by genetic polymorphisms of metabolic and DNA repair enzymes, are considered critical events alongside the theoretical pathway that links exposure to BC [5]. “Bulky” DNA adduct measurement has been therefore considered an integrated marker of both exposure to aromatic compounds and ability to activate carcinogens and repair DNA damage [6], [7]. Significantly higher levels of aromatic DNA adducts have been found in the bladder cancer biopsies from smokers [8], [9]. Moreover persistent aromatic-DNA adducts causing mutations, including mutational “hot spots” in the bladder P53 gene, has provided a solid mechanistic view on how DNA adducts may drive bladder tumourigenesis [10]. Furthermore, some DNA modifications induced by aromatic compounds in the bladder are found to mirror those in the peripheral blood lymphocytes (PBLs) [11], [12]. This has addressed the possibility of measuring such biomarker in accessible tissues which can be easily and non-invasively obtained from humans.

Many factors can however interfere in the theoretical pathway that links carcinogenic exposure to BC, such as multiple exposures (e.g., tobacco smoke, occupational exposure, fruit and vegetables consumption), their characterization (e.g., level, route, reliability) as well as the modulating role (increasing, protecting or having no effect) possibly played by polymorphic genes involved in metabolism and DNA repair [13]. To the best of our knowledge, only few studies have explored the hypothesis that PBLs DNA adduct levels can be associated to or predictive of BC risk. Results from three retrospective hospital based case-control showed that the risk indicator measuring the association between DNA adducts and BC was higher than 1.0 [14], [15], not different from unity [17], or lower than 1.0 [16]. More precisely, DNA adducts were associated to the risk of BC but independently from smoking habits [14], [15], while other authors [16] did not find any association between BC risk and bulky DNA adducts in never smokers. In the nested case-control prospective study, DNA adducts were not associated with BC risk [17]; overall, these conflicting results are hard to be explained from the biological viewpoint. Moreover, no study apparently estimated the complex interactions between DNA adducts, multiple genetic polymorphisms, occupational exposure to AAs and PAHs, and BC risk.

We previously assessed the interaction between occupational and environmental exposures with metabolic and DNA-repair polymorphisms on the risk of BC in retrospective hospital based case-control study [18]–[22].

The aim of this study was twofold: to investigate the extent to which PBL DNA adducts and BC risk were separately affected by genetic polymorphisms, environmental and occupational exposures; and to explore whether the formation of DNA adducts involved an additional increase in BC risk. These complex interrelationships were appraised using the analysis of structural equation modeling (SEM).

## Subjects and Methods

### Subjects

Study population, collection of data and statistical analysis are described in previous publications [18]–[22].

Briefly, the design was a hospital-based case-control study. The inclusion criteria were being male, aged between 20 and 80, resident in the Brescia province (Northern Italy). The cases were 201 newly diagnosed, histologically confirmed BC patients, admitted in the Urology Departments of the two main hospitals of Brescia from July 1997 to December 2000. The controls were 214 patients affected by various urological non-neoplastic diseases, frequency matched to cases by age (±5 years), period and hospital of admission. A written informed consent was obtained from each recruited subject and the study was approved by the the Spedali Civili di Brescia Ethical Committee.

All subjects were administered a questionnaire during hospital admission to collect information on demographic variables and lifetime history of smoking, coffee and other liquid consumption, diet habits, occupations. Occupational exposures to PAHs and AAs were estimated according to methodology described in previous publication [22]. An index of cumulative exposure to AAs and PAHs, separately, was calculated as product (i×f×l) of length (l), intensity (i) and frequency (f) of exposure in each job, summing up as many products as were necessary to take into account all jobs done. Life-long consumption of cigarettes was calculated as packyears. The lifelong time-weighted average of cups/day of coffee was recoded as 0 (never drinkers), ≤3, 4, ≥5 cups/day. PAHs containing food, fruit, large leaf vegetables and other vegetables consumption was divided into four categories (less than once/month; less than once/week; 1–3 times/week; more than 3 times/week).

Genotyping of GSTM1, GSTT1, GSTP1, NAT1, NAT2, SULT1A1, XRCC1-3, XPD, CYP1A2, MPO, COMT, MnSOD and NQO1 was assessed using Amplification Refractory Mutation System assay and using the GeneAmp PCR System 9700 (Applied Biosystems, Italy). PCR were followed by enzymatic digestion and PCR-RFLP analysis, as previously described [18]–[22].

All variables proved to be associated with BC risk at univariable logistic regression were forced in a multivariable unconditional logistic regression model and then chosen by backwards stepwise selection (with p<0.05 as criterion). BC risk significantly increased with packyears, heavy coffee drinkers, MnSOD (Val/Val genotype), while decreased with large leaf vegetables consumption and MPO (G-463A homozygous variant). Cumulative exposure to AAs was not statistically significant but it was retained because being a substantial confounder [19], [22].

### DNA extraction from PBLs

Blood samples were collected from all the subjects during hospital admission and on the same day processed by centrifugation for obtaining peripheral blood lymphocytes (PBLs). The protocol for automated DNA extraction was performed according to Extragen kit (Extragen BC. by TALENT) following the manufacturer's instructions as previously described [20]. In particular 2.5 ml of buffy coats prepared from up to 10 ml of whole blood were processed for DNA extractions. A typical yield ranged from 150 to 400 μg DNA/extraction from a normal donor.

^32^P-Post-labeling analysis of DNA adducts

Aliquots of 5 μg DNA were assayed for the presence of bulky-DNA adducts by ^32^P-postlabeling after enrichment with Nuclease P1 as previously described [23], [24]. Resolution of DNA adducts was performed by multidirectional thin-layer chromatography (TLC), using polyethyleneimine (PEI)-cellulose plates [25]. Briefly, 5 μg DNA were enzymatically digested to 3′-mononucleotides with 0,14 U/μg DNA of micrococcal nuclease and 1 mU/μg DNA of spleen phosphodiesterase for 3–4 hours at 37°C. After the enrichment procedure by Nuclease P1 digestion, DNA bases were labelled with 50 μCi of [gamma-^32^P]ATP with a specific activity of 5000 Ci per mmol by using 2.5 units of T4 polynucleotide kinase. 20 μl of postlabeled sample were spotted on the origin of a premarked PEI cellulose sheet and run for the multidirectional TLC chromatography. Following chromatography, TLC sheets were dried and electronic autoradiography performed using a ^32^P imager (InstanImager, Packard, MD, USA). A benzo(a)-pyrene diolepoxide-N2-dGp reference standard (National Cancer Institute Chemical Carcinogen Reference Standard Repository, Midwest Research Institute, Kansas City, Mo.) was used as a positive control in each labeling experiment. DNA adducts levels were measured as relative adduct level per 10^8^ nucleotides.

### Statistical analysis

In fitting SEM, packyears, coffee and vegetable consumption, *MnSOD*, *MPO*, cumulative AA exposure (variables associated with BC risk according to previous publications) plus *XRCC1* (see below) were used as exogenous variables (corresponding to predictors in regression based techniques). Both BC risk and adducts could be endogenous variables (corresponding to outcome variables), each affected by one or more exogenous variables (hypothesis 1). Alternatively, BC risk could be also influenced indirectly through the formation of DNA adducts (hypothesis 2). The two competing hypotheses were converted in two models of structural equations to find which model fitted best the observed data. SEM structural equations were fitted with “asymptotic distribution free” method because it did not make assumption on joint normality of all the variables and allowed using the variables (particularly adducts, see below) as given. The effect of each exogenous variable was expressed as standardized (or beta) coefficients that make comparisons easily by ignoring the independent variable's scale of units. SEM results were both tabulated and presented graphically. We used two SEM's goodness-of-fit statistics: (1) the chi square test for “model versus saturated” (the saturated model is the model that fits the covariances perfectly); and (2) the stability index obtained from the analysis of simultaneous equation systems.

The sample size required for SEM is dependent on model complexity, the estimation method used, and the distributional characteristics of observed variables [26]. The best option is to consider the model complexity (i.e., the number of exogenous variables) and the following rules of thumb: minimum ratio 5∶1 [27], [28]; recommended ratio 10∶1 [26]–[28]; recommended ratio 15∶1 for data with no normal distribution [29]. With eight exogenous variables used in the SEM model, we should have 120 ( = 15×8) subjects but they were actually 412 (see below) fulfilling the above requirements.

The analysis was carried out with the statistical package STATA 12.

## Results

In the present study, complete individual data were available for 199 (out of 201) cases and 213 (out of 214) controls, totaling 412 (instead of 415) subjects.

Table 1 shows the main characteristics of cases and controls. Current and former smoking were more common (chi^2^ test (2df)  = 32.2377; p = 0.000) and coffee intake was higher (Wilcoxon rank-sum test z = −3.756; p = 0.0002) in cases than in controls. No significant differences between cases and controls were found for other demographic variables and putative risk factors of BC.

**Table 1:** Distribution of demographic characteristics, life habits, and occupational exposures in cases and controls.

	Cases (n = 199)	Controls (n = 213)
	Number (Percentage)	Number (Percentage)
Age		
≤45	14 (7)	19 (9)
46–55	25 (13)	28 (13)
56–65	58 (29)	76 (36)
66–75	82 (41)	69 (32)
>75	20 (10)	21 (10)
Education		
0–5	106 (54)	111 (53)
6–8	60 (30)	47 (22)
9–13	25 (13)	41 (20)
≥14	7 (4)	11 (5)
Lifetime smoking		
Never	17 (9)	54 (25)
Light (≤26 packyears)	56 (28)	78 (37)
Heavy (>26 packyears)	126 (63)	81 (38)
Occupational cumulative exposure to PAHs		
Never	128 (64)	143 (67)
Ever	71 (36)	70 (33)
Occupational cumulative exposure to AAs		
Never	182 (91)	203 (95)
Ever	17 (9)	10 (5)
Coffee consumption (cups/day)		
Mean (± Standard Deviation)	2.33 (±2.30)	1.58 (±1.62)
5th–95th percentiles	0–8	0–6

DNA adduct levels ranged from 0.3 to 70 adducts ×10^8^ nucleotides; the variable did not follow a normal distribution and any transformations failed to reduce its skewness (data not shown). Table 2 shows that mean, standard deviation, median, interquartile range and CV% of adduct levels were similar in cases and controls. A multivariable linear regression model with backwards stepwise selection gave *XRCC1* as the only significant variable carrying protective effect (data not shown).

**Table 2:** Summary statistics (mean, standard deviation, median, interquartile range, number of subjects) for “ln_adducts” in cases, controls, and total population.

	Mean	Std. Dev.	CV%	Median	Inter quartile range	N
Cases	0.82	1.20	146	0.57	2.00	185
Controls	0.77	1.09	142	0.46	1.73	180
Total	0.80	1.14	143	0.51	1.83	365

Table 3 shows three groups of SEM results.

**Table 3:** SEM results: beta coefficients (with “minus” sign indicating inverse relationship), standard errors, z tests and the corresponding p-values, along with 95% confidence intervals for endogenous variables of structural equations; variances and covariance.

	Endogenous variable	Exogenous variables	Beta Coef.	Std. Err.	z	P>|z|	95% CI	
							Lower	Upper
Structural Equations	Adducts	Packyears	0.064	0.048	1.35	0.177	−0.029	0.157
		Coffee	0.015	0.047	0.31	0.757	−0.078	0.107
		Occupational exposure to AAs	0.117	0.048	2.46	0.014	0.024	0.210
		XRCC1	−0.129	0.047	−2.75	0.006	−0.221	−0.037
		MPO	−0.024	0.039	−0.63	0.531	−0.100	0.052
		MnSOD	−0.053	0.045	−1.17	0.244	−0.142	0.036
	Cancer Risk	Packyears	0.256	0.041	6.19	0.000	0.175	0.337
		Coffee	0.166	0.043	3.83	0.000	0.081	0.250
		AA	0.084	0.034	2.45	0.014	0.017	0.152
		XRCC1	−0.028	0.044	−0.65	0.519	−0.114	0.058
		MPO	−0.115	0.036	−3.21	0.001	−0.185	−0.045
		MnSOD	0.120	0.044	2.71	0.007	0.033	0.206
Variances	Adducts		0.962	0.018			0.927	0.998
	Cancer Risk		0.858	0.025			0.811	0.908
Covariance	Adducts × Cancer Risk		−0.038	0.047	−0.80	0.421	−0.131	0.055

Packyears  =  Life-long consumption of cigarettes.

Coffee  =  Life-long time-weighted average of cups/day of coffee.

AA  =  Occupational cumulative exposure to aromatic amines.

*XRCC1*  =  X-ray repair cross-complementing protein 1.

MPO  =  Myeloperoxidase (*G-463A* homozygous variant).

*MnSOD*  =  Manganese Superoxide Dismutase (*Val/Val* genotype).

1. Structural equations. It can be seen the beta coefficients (with “minus” sign indicating an inverse relationship), standard errors, z tests with p-values, and 95% confidence intervals for each of two structural equation models. The first model shows that cumulative occupational exposure to AAs (beta  = 0.117; p = 0.028) is associated with increased DNA adduct levels, whereas *XRCC1 Arg 399* (beta  = −0.129; p<0.006) with decreased levels. We calculated the corresponding study-wise p-value as (1-(1-alpha)^n^), where “alpha” was 0.006 and “n” was 6. The result was 0,035464301 (well below the threshold of statistical significance), indicating that the error probability of 0.006 cannot be an effect of random fluctuations. The second model shows that cigarette smoking (packyears; beta  = 0.256; p<0.001), coffee (beta  = 0.166; p<0.001), cumulative occupational AAs exposure (beta  = 0.084; p = 0.041) and *MnSOD* (beta  = 0.119; p = 0.009) increased the BC risk whereas *MPO* (beta  = −0.115; p<0.008) decreased it. No indirect paths were demonstrated (disproving hypothesis 2).
2. Variance explained by the above fitting was about 4% for DNA adducts and roughly 14% for the risk of BC.
3. Covariance between the two endogenous variables (BC risk and adducts) was not significant (p = 0.441, last row of table 1). This finding demonstrated that these variables were not correlated to each other and that supported the hypothesis 2, i.e., DNA adducts did not affect BC risk in our population.

The value of chi square test for the discrepancy of the specified model versus saturated model was 0.00 with p-value equal to 1.00. The stability index was 0.00, indicating that SEM satisfies stability condition.

**Figure 1:**
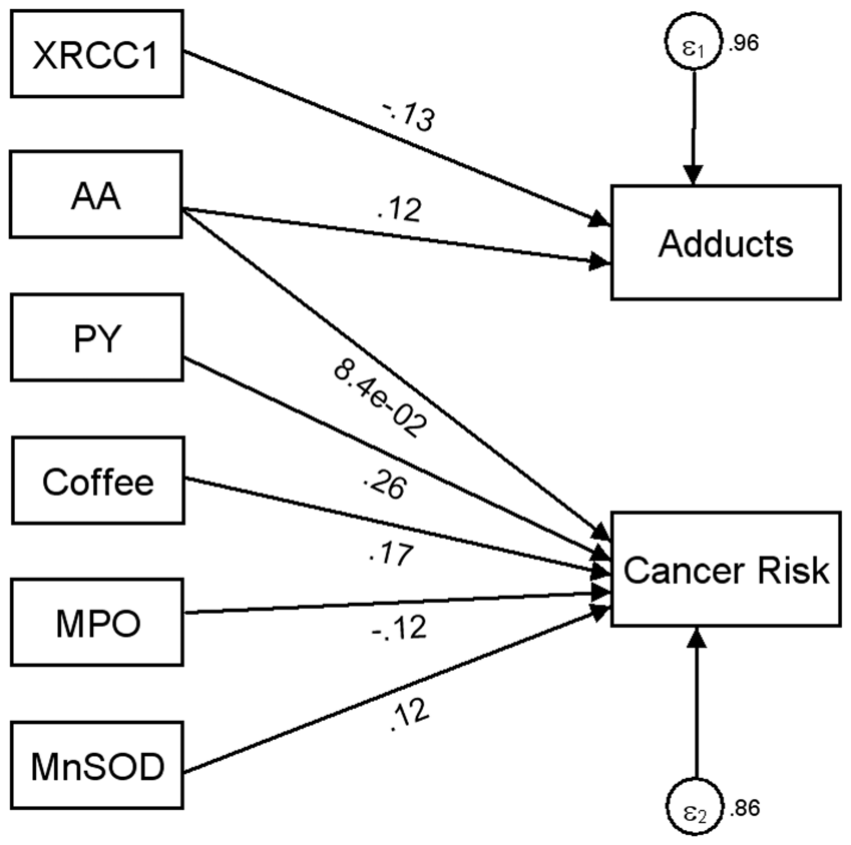
Path diagram of results shown in table 1. Variables (square boxes); variances (circles); causal flow (arrows); and paths (arrowed route). The estimated beta coefficients appeared along the paths. LEGEND: Packyears  =  Life-long consumption of cigarettes; Coffee  =  Life-long time-weighted average of cups/day of coffee; AA  =  Occupational cumulative exposure to aromatic amines; *XRCC1*  =  X-ray repair cross-complementing protein 1; *MPO*  =  Myeloperoxidase (G-463A homozygous variant); *MnSOD*  =  Manganese Superoxide Dismutase (Val/Val genotype).

Using the graphical interface of SEM, the results shown in table 3 were displayed as path diagram in figure 1. In this figure, square boxes stand for variables, circles indicate variances, arrows specify the direction of causal flow, an arrowed route is a path, and the estimated beta coefficients appeared along the paths. The effect of one variable on another is called direct. There was no evidence of indirect effect (one variable affecting another variable which in turn affects a third), indicating that bladder cancer risk in our population was not further increased through formation of DNA adducts.

## Discussion

### DNA adducts as outcome

Since DNA adducts in PBLs are considered an integrate measure of carcinogen exposure, absorption, distribution, metabolism, and DNA repair, they have been referred to as biomarker of ‘biologically effective dose’ i.e. a measure of the amount of the carcinogen at the critical target [13]. Moreover carcinogen DNA adduct levels in more accessible circulating PBLs mirror those in the bladder tissue [11], [12], which are considered an initial step in carcinogenesis.

However, in the present study, consistent with other literature findings, bulky DNA adducts detected by the nuclease P1 method of ^32^P-post-labeling were not associated with an increased BC risk, probably because the adducts measured in PBLs at the time of BC diagnosis represent snapshots that are not necessarily representative of exposures relevant for BC risk that occurred in the past.

Adduct levels were instead associated with *XRCC1399Arg* carriers who presented a significant reduction in DNA adducts level, but with no effect on the risk of BC. Our results agree with previous studies reporting *XRCC1399Arg* associated with lower levels of bulky DNA adducts [30], [31], due to higher DNA repair activity [32], and with two recent meta-analyses where *XRCC1 Arg399Gln* polymorphism was not related to BC risk [33], [34].

In the present study, likewise the above reported studies [14], [15], [17], no relationship between DNA adduct levels and smoking was reported. Previous ^32^P-postlabeling studies have reported inconsistent results on the association between the adduct levels in PBLs and tobacco smoking [35]–[39]. Discrepancies may depend on the marked interindividual variation in the metabolism of smoking carcinogens, which results in different DNA adduct levels for similar degrees of exposure [40]. Moreover, most of studies on the effect of smoking on PBLs bulky-DNA adduct levels did not reveal any significant differences [35], [36], [39], suggesting that adducts in PBLs from smokers may result from sources other than tobacco smoking.

We also found an association between cumulative occupational exposure to AAs and PBL adduct levels that was instead not reported by other authors most probably because information about occupational exposures was too limited to allow evaluation, even in the largest case-control study nested in EPIC cohort [17].

### BC risk as outcome

We confirmed by SEM analysis the biological plausible protective effect of *MPO A/A* [21], and the risk effect of *MnSOD Val/Val* on BC [21], [41]. The genetic polymorphism of enzymes involved in individual response to oxidative stress is likely involved in modulating the individual response to environmental exposures such as tobacco smoking, coffee drinks and AAs exposure [21]. In this study we also confirmed our previous results on the association between smoking habit and coffee drinking on BC risk, where the latter might be attributed to residual confounding by inadequate adjustment for cigarette smoking (which is over-represented among those who drink the most coffee/caffeine) [22]. As shown in table 3, the level of statistical significance of beta coefficients was particularly high for the association between BC risk and, on the other hand, packyears (beta  = 0.256; p<0.001) and coffee (beta  = 0.166; p<0.001); as well as for the association between decreased DNA adduct and *XRCC1 Arg 399* (beta  = −0.129; p<0.006). Despite this, the proportion to which SEM fitting accounts for the dispersion of data (variance explained) was as low as 16% for BC risk, and 4% for DNA adducts. Therefore most variation of outcomes should be attributed to unknown predictors.

With regard to the relationship of occupational exposures to AAs and PAHs, DNA adducts and BC risk the literature is scanty and those few published studies have only found an exposure-independent association between adducts and BC risk. Our study precisely evaluated the occupational exposure history to AAs and PAHs, and noted a significant correlation between occupational exposure to AAs and BC, and adduct levels too. Therefore occupational exposure to AAs is confirmed as central risk factor for BC development. Finally the present work was carried out in the context of a biologically plausible and hypothesis-driven study design consistent with the available literature data.

In addition, the correlation we found between AAs exposure and BC risk is biologically plausible, because AAs are activated in liver and transported by blood proteins to the bladder where, under acidic conditions [42], [43] or, enzymatically by O-acetylation of N-hydroxy arylamine (predominantly by the N- acetyltransferase 1 (NAT1) isozyme), are further activated to the ultimate carcinogen [44]. This result also suggests that occupational exposure history collected by questionnaire through interview could be a reliable measurement of exposures to AAs in such studies.

Unlike AAs, cumulative exposure to PAHs was not associated with BC risk. Experimental evidence suggests that PAHs are slowly absorbed through most tissues. For instance, in the case of dermal exposure, considered the main route in the industry [45], absorption accounts for a small fraction of applied dose, and PAHs are enzymatically activated and degraded at this site of entry [46]–[48]. The concentration and persistence of PAHs in the lung is largely related with inhalation of PAHs containing dust [49], [50]. The high propensity of PAHs to act as carcinogens at the sites of entry is supported by several experimental studies [51].

### DNA adducts and BC risk

The results of literature on the association between DNA adducts and risk of BC are scanty and not consistent. A strong direct association between and the risk of BC and PBLs DNA adducts, measured at time of diagnosis and detected by means nuclease P1 and ^32^P-postlabeling, was reported in two retrospective hospital based case-control [14], [15]. The association was however independent from smoking habits and was suggested to be dependent from other exposures (that the studies did not specify), and from *NAT2* as genetic factor. While, in a retrospective hospital based case-control among nonsmokers bulky DNA adducts were not associated with bladder cancer risk [16]. Lastly PBLs DNA adducts (nuclease P1 and 32P-postlabeling) were not associated with the subsequent bladder cancer insurgence in a prospective nested case-control study [17]. The findings of this latter study are probably more reliable and meaningful than those of earlier investigations [14], [15], because DNA adducts were measured years before the onset of disease, thus ruling out the possibility that the higher adduct levels were due to a condition associated with an already existing cancer.

We did not find any relation between DNA adducts and BC risk. However, we cannot exclude such relationship. In fact, a limitation of the present study is that adducts measured by nuclease P1 method of ^32^P-postlabeling are non-specific because the responsible electrophilic substance cannot be identified. Moreover, DNA adducts measure at time of diagnosis may be not representative of cumulative doses of carcinogens that may cause cancer. Environmental and occupational exposures vary both qualitatively and quantitatively over time due to changes in lifestyle, place of residence, employment, etc., and the impact of a given exposure on the risk of cancer cannot be constant throughout the life of an individual [13]. This fact, combined with the awareness that the rate and speed of repair of various DNA adducts are different and their permanence mainly depends from life span of PBLs, from a few days to a few weeks, poses uncertainty on the significance of such short-term exposure biomarker in relation to the risk of cancer. Given the long latency of carcinogen-related malignancies – that is the time between the beginning of exposure and the onset of disease – the retrospective assessment of carcinogen exposures, especially via biomarkers, represents a challenge for both epidemiology and clinical medicine.

### Perspectives (including SEM analysis)

New opportunities for biomonitoring of carcinogens may derive from measuring exposures from all sources both external and internal that occur throughout the lifespan. This approach named exposome by Wild [52] is represented by the set of chemicals derived from sources outside genetic control that include diet, pathogens, microbiome, smoking, psychological stress, drugs and pollution [53]. Indeed, these new technologies are opening new scenarios for biomarker discovery but new challenges as well, that include the need of repeated measurements of global sets of biomarkers to be collected at different critical life stages. Only in this way can the dynamics of exposures and early and late effects be captured.

In medicine and natural sciences a given outcome is often affected or influenced by more than one thing simultaneously. Multivariate techniques try to statistically account for these differences, adjusting an outcome measure Y to a 1 unit change in X, holding all other variables constant. However, it may be that other variables are not likely to remain constant: a change in X can produce a change in Z (direct effect) which in turn produces a change in Y (indirect effect). Both the direct and indirect effects of X on Y must be considered if we want to know what effect a change in X will have on Y. This can be done mathematically and statistically only using SEM. The procedure decomposes a correlation between two variables into its component parts: direct effects, indirect effects, common causes (X affects both Y and Z; this is spurious association) and correlated causes (X is a cause of Z and X is correlated with Y). The user is required to state, often using a path diagram, the way that he/she believes the variables are inter-related. Via some complex internal rules, SEM decides which model fits data better. This method is more suitable to analyze complex interrelationships because it tests causal relationships rather than mere correlations.

In our opinion, the statistical analysis with SEM is one strength of the present research. Other strengths of the study are the thorough and reliable collection of several personal, occupational and environmental variables, the multiple genetic polymorphisms and endpoints, the significant number of subjects, as compared to other similar studies with adduct analysis [14]–[17], the quality of DNA adduct analysis, the sample size required for calculation of SEM; here, the actual number of 412 cases was much higher than the maximum required sample size of 120 subjects estimated according to different assumptions (see above: statistical analysis).

## Conclusions

Using the SEM analysis, a statistical technique that combines observed data and qualitative causal assumptions and tests whether and how variables are interrelated through a system of equations, we found that PBL DNA adducts was not associated with BC risk. This suggests that this measure at time of diagnosis may be not representative of dose of carcinogens that may cause cancer. However the new finding stemming from this study sustains that occupational cumulative exposure to AAs were instead associated with both DNA adducts and with BC risk. This agrees with the propensity of AAs to act as carcinogens away of the sites of entry after being transported by blood proteins to the bladder and confirm exposure to AAs, determined by blood DNA adduct, as central risk factor for BC development. Moreover *XRCC1399Arg* polymorphism has a role in repairing PBL DNA adducts but no impact on individual susceptibility to BC. Previous findings on the influence of smoking, coffee intake, *MPO A/A* and *MnSOD Val/Val* polymorphisms on BC risk were also confirmed by SEM analysis. A direct effect of these predictor variables was observed on each outcome variable. Our study envisages new scenarios, entailing the need of repeated DNA adduct measurements at different critical life stages and proper analytical techniques, for example during occupational exposure to AAs, as well as the appraisal of the complex relationship between gene and environment, by means of SEM analysis.
